# Strength of sliding knots in multifilament resorbable suture materials

**DOI:** 10.1007/s10397-012-0753-5

**Published:** 2012-06-16

**Authors:** Nourah van Leeuwen, J. Baptist Trimbos

**Affiliations:** Department of Gynecology, Leiden University Medical Center, POB 9600, 2300 RC Leiden, The Netherlands

**Keywords:** Knots, Sliding knots, Knot reliability, Sutures, Resorbable multifilament sutures

## Abstract

Experimental laboratory study was made to test the knot integrity of identical, non-identical and parallel sliding knots, with three and five throws, made with synthetic resorbable multifilament suture materials. The knots were made with Novosyn (polyglactin 612), Safil (polyglycolic acid), Vicryl (polyglactin 612) and Vicryl plus (polyglactin 910 + triclosan), all with suture size: 3-0 USP. Per material 10 knots for every kind of knot were tested in a tensiometer, resulting in a total of 240 tests. Sliding knots with three throws were compared with the five throw sliding knots, and a comparison of the loop-holding capacities (LHC) of the different suture materials was made. Differences in suture material, knot type, and number of throws in the knot had a remarkable effect on knot performance. Adding two extra throws to a three throw non-identical or parallel sliding knot resulted in significantly more reliable knots (*P* < 0.05). In identical sliding knots, this effect was not seen, but these knots showed low LHCs, indicating poor knot reliability. Compared to the other suture materials, Safil showed significantly lower LHCs. Most of the mean LHCs of the various knots with Vicryl, Vicryl Plus or Novosyn were not statistically different from each other. Identical sliding knots appeared to be very unreliable, especially when made with three throws. Non-identical and parallel slipknots with five throws demonstrated superior knot integrity compared with the same knot types with three throws. Safil had inferior knot properties as compared to the other materials, but Vicryl, Vicryl Plus and Novosyn behaved virtually the same. The type of knot and the use of different suture materials have important influence on the integrity of the knot. A high knot reliability is nowadays all the more important because of the frequent use of resorbable suture materials. The suture gradually loses strength during the resorption process, so that an extra margin of safety neutralizes the effect of this process.

## Introduction

The choice for a particular suture material and knot type is frequently a matter of personal preference of the surgeon or hospital tradition [1], but using a specific material or knot can have great consequences for the patient. Breakage or slippage of a suture can lead to serious complications. Wound dehiscence, incisional hernia or internal haemorrhage may derive from knot failure in the abdominal wound [2]. Therefore, the holding power of a knot should, next to knot bulk, tissue reaction and handling properties, be considered an important factor in electing a particular suture material [3]. Nevertheless, to date, only few studies investigating the properties of suture materials and knots have been published. The studies that have been published often deal with older or even out-of-date suture materials.

Although the flat square knots are considered very secure with low failure rates across a variety of suture materials [4], sliding knots are the most frequently used in surgery. Gynaecologic surgeons also prefer sliding knots, because of the advantage that one suture end can be kept under constant tension while tying in the narrow deep spaces of the vagina and the pelvis. Therefore this study examined the performance of different types of sliding knots made with modern, resorbable suture materials.

## Materials and methods

**Fig. 1:**
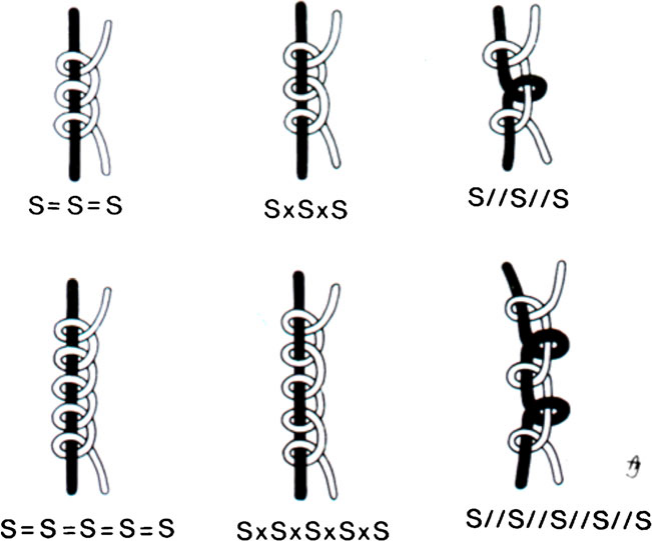
Configuration and code of the six different knots studied. Sliding throws (*S*), identical throws around the same suture (*equals sign*), nonidentical or crossed sliding throws around the same suture (*multiplication sign*), sliding throws alternately tied around different sutures (*double solidus*)

Sterile suture material, intended for operating room use, was obtained from the hospital’s stock. The tested synthetic resorbable multifilament suture materials were: Novosyn (polyglactin 612), Safil (polyglycolic acid), Vicryl (polyglactin 612) and Vicryl Plus (polyglactin 910 + triclosan). In all cases suture size 3-0 United States Pharmocopeia (USP) was tested. The analysed knots were: identical, non-identical and parallel sliding knots, all with three and five throws. To describe the different knots, a code system was used that was described previously [5]. Figure 1 shows the codes of the tested knots and the corresponding configuration. Identical sliding knots were made by repeating the same tying action with the same hand, e.g. backhand or forehand. Non-identical sliding knots were made by alternating backhand and forehand tying with the same hand. Parallel sliding knots were made by changing the suture thread that was kept under tension and alternately tying the suture with the left and right hand.

Per material 10 knots for every kind of knot were tested, resulting in a total of 240 tests. After soaking for 15 min in saline, the suture materials were tied around two elliptical rods attached to a board. The knots were carefully tied by one of the authors (JBT), and the type was verified before the knot was tied tight. Then the tension on the loop was removed by rotating one of the rods, so that the suture loop could be removed. Blinded to the tested suture material and knot type, the other author (NvL) placed the loop over two polished metal axles in a tensiometer. The axles moved apart at a constant rate of 25 mm/min and caused increasing tension on the loop. The strength by which failure of the loop occurred was registered to define the “loop-holding capacity” (LHC): the force required to break the suture or provoke slippage in the knot [6]. This measure was registered in newtons and used in the further analysis of the experiments. The way of knot failure was noted to determine whether certain knot types failed more frequently due to slippage or breakage.

## Results

In 186 of the 240 tests, knot failure was due to slippage of the knot: 73.3 % (*N* = 44) in the Novosyn group, 85.0 % (*N* = 51) in the Safil group, 71.1 % (*N* = 43) in the regular Vicryl group and 80.0 % (*N* = 48) in the Vicryl Plus group. In the remaining 54 tests, the suture broke in the knot or in the immediate vicinity of the knot.

Table 1 shows the mean LHCs with standard deviation for each type of knot. A comparison of the LHCs of the different suture materials is shown in Table 2. In the comparison of regular Vicryl and Safil, Vicryl showed superior knot profiles. The only exception was in the three throw identical sliding knot. Besides for the identical slipknot with five throws (S=S=S=S=S) the comparison of Vicryl and Novosyn showed no significant differences in all the knot types. Novosyn sutures were more reliable than Safil in four of the six knots tested. Vicryl Plus showed the same knot performance as compared to regular Vicryl.

**Table 1:** Mean loop-holding capacity with standard deviation as determined in six different kinds of slipknots, for each type of suture material

Knot type	Suture material			
	Vicryl plus	Vicryl	Safil	Novosyn
S=S=S	5.3 ± 0.95	7 ± 5.73	3.1 ± 2.08	4.8 ± 1.75
S×S×S	21.5 ± 11.79	18.8 ± 9.77	9.5 ± 4.06	22.9 ± 14.56
S//S//S	29.8 ± 9.87	35.8 ± 15.38	21.0 ± 6.02	43.4 ± 17.33
S=S=S=S=S	11.4 ± 4.90	10.8 ± 1.93	3.2 ± 1.93	4.2 ± 4.13
S×S×S×S×S	28.7 ± 5.25	34.0 ± 15.52	18.6 ± 6.52	37.6 ± 11.05
S//S//S//S//S	57.6 ± 3.81	58.4 ± 2.32	50.4 ± 7.35	59.3 ± 2.87

**Table 2:** Statistical analyses of differences in loop-holding capacities between the different suture materials

Knot type	Compared suture materials			
	Vicryl plus vs. Vicryl regular (*P* value)	Vicryl vs. Safil (*P* value)	Vicryl vs. Novosyn (*P* value)	Novosyn vs. Safil (*P* value)
S=S=S	*0.939*	*0.146*	*0.675*	*0.059*
S×S×S	*0.820*	0.001	*0.161*	0.006
S//S//S	*0.256*	0.011	*0.384*	0.002
S=S=S=S=S	*0.702*	<0.001	0.003	*0.908*
S×S×S×S×S	*0.704*	0.014	*0.449*	<0.001
S//S//S//S//S	*0.675*	0.013	*0.401*	0.003

Values in upright represent a statistical significant difference, determined by a Mann–Whitney test over the two samples. Values in italics represent statistically nonsignificant differences

Sliding knots with three identical throws around the same suture (S=S=S) appeared to be very unreliable (Table 1). The adding of two extra throws resulted in a significantly more secure knot only in the Vicryl Plus group (Table 3). All the non-identical slipknots with five throws (S×S×S×S×S), irrespective of the suture material, showed significantly higher LHCs compared with the same knot types with three throws (S×S×S; Table 3). The comparison of parallel slipknots with three (S//S//S) vs. five throws (S//S//S//S//S) displayed the same, except for the Safil group (Table 3).

**Table 3:** Statistical analyses of differences in loop-holding capacity between three and five throw slipknots, per suture material

Suture material	Compared knot types		
	S=S=S=S=S vs. S=S=S (*P* value)	S×S×S×S×S vs. S×S×S (*P* value)	S//S//S//S//S vs. S//S//S (*P* value)
Vicryl plus	<0.001	0.034	<0.001
Vicryl (regular)	*0.111*	0.008	<0.001
Safil	*0.908*	0.004	*0.053*
Novosyn	*0.234*	0.005	<0.001

Values in upright represent a statistical significant difference, determined by a Mann–Whitney test over the two samples. Values in italics represent statistically nonsignificant differences

## Discussion

**Fig. 2:**
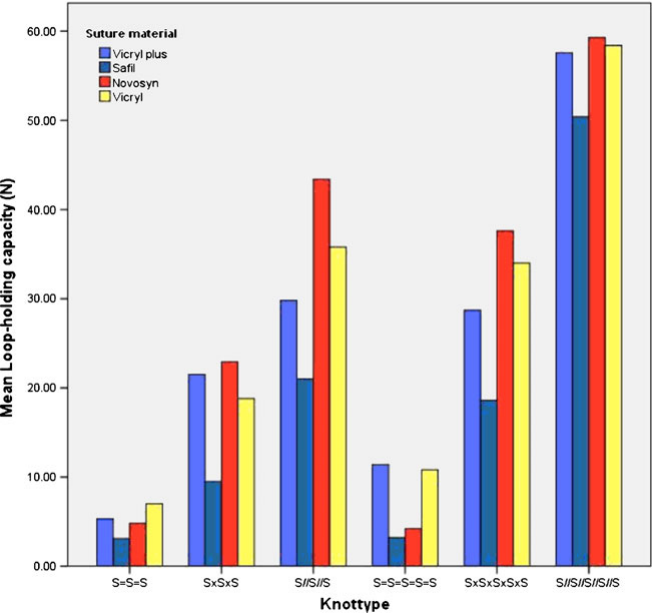
Mean loop-holding capacities per material clustered by knot type

This study shows remarkable differences in knot strength between different sutures, knot types and numbers of throws in the knot (Fig. 2). The identical sliding knots with three throws (S=S=S) appeared to be very unreliable. When two identical throws were added (S=S=S=S=S), the difference was only significant in the Vicryl Plus group. All the non-identical slipknots with five throws (S×S×S×S×S) showed significantly higher LHCs compared with the same knot types with three throws (S×S×S). The parallel sliding knots displayed the same, except for the Safil group. This discrepancy between sutures of the effect of adding two extra throws to the knot might be explained by the finding that even five throw identical sliding knots show poor knot reliability. In other words, a change from very poor to poor is still not good enough. The consequence of this is that the application of identical sliding knots should be discouraged in clinical practice and this confirms earlier findings of this kind [6].

On the evidence of these experiments, there is no significant difference between regular Vicryl and Vicryl Plus. Apparently, the adding of an aseptic compound to the suture to prevent wound infection has no demonstrable effect on the friction coefficient of the suture and its knot reliability. The comparison of Vicryl and Safil showed superior results for Vicryl in all knot types tested, except for the identical sliding knot with three throws (S=S=S). The comparison of Vicryl and Novosyn showed no differences, except for the identical slipknot with five throws (S=S=S=S=S) in which Vicryl did better. All the knot types showed higher LHCs with Novosyn in comparison with Safil, besides the identical slipknots with three and five throws (S=S=S and S=S=S=S=S). These findings indicate that Safil has inferior knot performance as compared to Vicryl and Novosyn. Novosyn and Vicryl showed similar knot profiles.

Our results comply with former investigations [7]. In a more recent study, Ivy et al. compared the knot integrity of non-identical sliding knots with three and six throws, made with polydioxanone and polyglactin 910 sizes 0 and 2 USP. This study also showed that an increasing number of throws resulted in significantly more reliable knots and decreasing knot failure due to slippage, irrespective of the suture type or gauge [8].

There is still little knowledge about the exact forces that a knotted suture in the human body must be able to tolerate. These forces probably differ among individuals, they differ among the type of tissue they are in and they are liable to many local factors. Until further knowledge is acquired, it seems advisable to ensure that a knotted surgical suture is at least as strong as the tissue it surrounds [1]. The surgeon can improve the strength of a knotted suture loop in different ways. Changing the suture gauge is one way [9], and choosing a suture material with better knot profiles is another. As shown in this study, the surgeon can also add more throws to the knot. And last but not least, the surgeon can change to another type of knot that is more reliable. This has been an important vision for years, but nowadays, it is all the more important because of the frequent use of resorbable suture materials. During the resorption process, the suture gradually loses strength. A high knot reliability is therefore even more important, in that an extra margin of safety neutralizes the effect of this process. Every surgeon should have knowledge of the differences in knot security between the variable suture materials and knot types, and should strive to make the most secure knot, with the ideal suture material for the task and a minimal amount of foreign body suture material.
